# Increased impact of heat domes on 2021-like heat extremes in North America under global warming

**DOI:** 10.1038/s41467-023-37309-y

**Published:** 2023-03-27

**Authors:** Xing Zhang, Tianjun Zhou, Wenxia Zhang, Liwen Ren, Jie Jiang, Shuai Hu, Meng Zuo, Lixia Zhang, Wenmin Man

**Affiliations:** 1 State Key Laboratory of Numerical Modeling for Atmospheric Sciences and Geophysical Fluid Dynamics (LASG), Institute of Atmospheric Physics, Chinese Academy of Sciences, Beijing, 100029 China; 2 University of Chinese Academy of Sciences, Beijing, 100049 China; 3 China Meteorological Administration, Beijing, 100081 China

**Keywords:** Climate change, Climate-change impacts

## Abstract

During summer 2021, Western North America (WNA) experienced an unprecedented heatwave with record-breaking high temperatures associated with a strong anomalous high-pressure system, i.e., a heat dome. Here, we use a flow analog method and find that the heat dome over the WNA can explain half of the magnitude of the anomalous temperature. The intensities of hot extremes associated with similar heat dome-like atmospheric circulations increase faster than background global warming in both historical change and future projection. Such relationship between hot extremes and mean temperature can be partly explained by soil moisture-atmosphere feedback. The probability of 2021-like heat extremes is projected to increase due to the background warming, the enhanced soil moisture-atmosphere feedback and the weak but still significantly increased probability of the heat dome-like circulation. The population exposure to such heat extremes will also increase. Limiting global warming to 1.5 °C instead of 2 °C (3 °C) would lead to an avoided impact of 53% (89%) of the increase in population exposure to 2021-like heat extremes under the RCP8.5-SSP5 scenario.

## Abstract (web summary)

The heat dome explains about 55% of the 2021 Western North American high temperatures. The intensity of heat extremes associated with such circulations are increasing faster than background global warming due to soil moisture feedbacks.

## Introduction

Climate extremes such as heatwaves, heavy rainfall and drought are of great concern to policy-makers due to their profound impact on society, the economy and human lives^1,2^. Since the 1950s, extreme events have been increasing in intensity and frequency across the globe, with the most significant changes occurring in hot extremes (including heatwaves)^3^. Record-breaking heatwaves have been researched foci of the climate change community, such as the hottest summer in Australia from December 2012 to February 2013^4^, the July August 2013 heat event in Central and Eastern China, Japan and Korea^5–8^, the June 2017 mega-heatwave in western and central Europe^9^, and the unprecedented heatwaves over Northeast Asia in summer 2018^10,11^. During the last week of June 2021, an extraordinary heatwave affected Western North America (WNA, including the northwestern United States and western Canada) and had a daily maximum temperature above 40 °C (Supplementary Fig. 1). The daily maximum temperature anomalies reached 16  °C to 20  °C in many North American cities^12^. The event was one of the most extreme heat events recorded around the world since 1950^13^. Over 1400 people lost their lives as a result of the heatwave and numerous wildfires occurred during and after the event^14–16^.

Heat events are closely associated with atmospheric circulation anomalies, which typically present as an anomalous high-pressure system^17–19^. These systems maintain heatwaves in an area through enhanced insolation, the transport of warm and moist air from lower latitudes, and reinforced subsidence^20^. Using the *flow analog method*^21^, the contribution of atmospheric circulation to extreme events such as heatwaves and heavy precipitation can be quantified^22–25^. For example, the stable persistence of anomalous anticyclones played an important role in the record-breaking heat event that occurred over Northeast Asia in summer 2018, explaining half of the magnitude of the observed temperature anomalies^24^. The change in atmospheric circulation, i.e., dynamical changes, also has an impact on extreme events^26,27^.

A high-pressure system called a “heat dome”, trapping hot ocean air like a cap^28^, was a key dynamic component of the summer 2021 heatwave over the WNA^15,29^. Rapid attribution concluded that human-caused climate change increases the probability of extreme heatwave by at least 150 times^12^. The observed and projected increases in the heat events are in line with the mean background warming^3,13^. However, there is still a lack of quantitative estimation of the contribution of atmospheric circulation to the unprecedented heatwave over the WNA. More importantly, how the heat dome has and will continue to affect such a heatwave remains unknown. Here, based on the flow analog method, we quantify the contribution of the heat dome to the extreme heat event over the WNA and investigate the influence of background warming on the heatwave. We demonstrate that the heat dome explains over 50% of the magnitude of the observed heatwave. The background warming and the soil moisture-atmosphere feedback would be more conducive to the occurrence of heatwaves under a warmer climate.

## Results

### Observed characteristics of the 2021 heatwave in Western North America

**Fig. 1:**
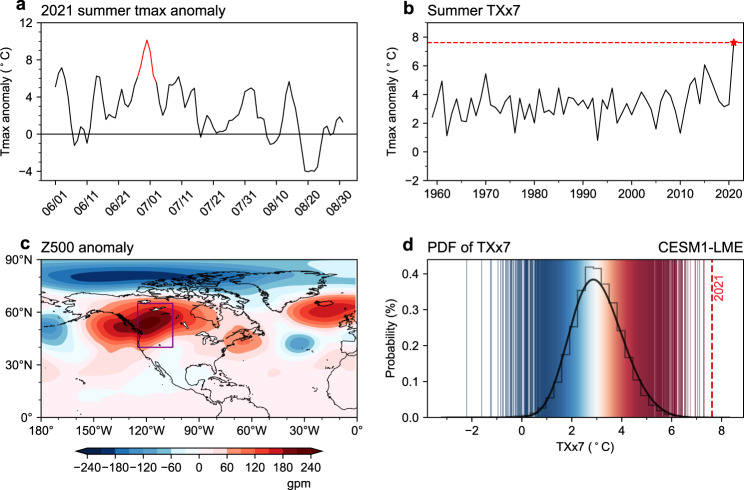
The characteristics of the heatwave of summer 2021 over the Western North America (WNA, 40°−65°N, 125°−105°W). **a** Evolution of maximum temperature anomalies (relative to 1981-2010, unit:  °C) in summer 2021 area-weighted averaged over the WNA (purple box in Fig. 1c). **b** Evolution of the anomalies of *TXx7* (unit:  °C) area-weighted averaged over the WNA during 1959–2021. *TXx7* is defined as the annual summer (June–August) maxima of the 7-day running mean of the daily maximum temperature anomaly area-weighted averaged over the WNA. The red line indicates the *TXx7* anomalies (27 June–3 July) for 2021. **c** The geopotential height anomalies (shading, unit: gpm) at 500 hPa for 27 June–3 July 2021. **d** The probability density functions (PDF) of the corrected *TXx7* (see Methods) averaged over the WNA during 850–2005 for the 12-member realization of CESM1 last millennium ensemble (LME) simulation. The red dotted line indicates the *TXx7* of 2021 from ERA5.

We first examine the characteristics of the heat event, including circulation and temperature. On 27 June-3 July 2021, the average daily maximum temperature (tmax) anomalies of the WNA (40°−65°N, 105°−125°W) were highest of that summer, peaking on 30 June with a temperature anomaly of 10.13 °C relative to the 1981–2010 climatology (Fig. 1a). The *TXx7* index, which is defined as the annual summer (June-August) maxima of the 7-day running mean of daily maximum temperature anomaly area-weighted averaged over the WNA (see Methods), has exceeded the 1981–2010 climatology by ~5 standard deviations ($\sigma$) in 2021 and broke the records since 1959 (*TXx7* in 2021: ~7.61  °C) (Fig. 1b), indicating an exceptional week-long heatwave in summer 2021. The heat dome, viz. the anomalous anticyclone was steadily located over the WNA during the heat event (Fig. 1c and Supplementary Fig. 2), which is the dominant circulation system for the extremely high temperature.

We extend the analysis to the last millennial climate simulation (see Methods). In the context of historical climate simulations (Fig. 1d), the temperature of 2021 was hottest in the last millennium (850–2005), indicating that the summer 2021 heat event was unprecedented for the model world.

### Contribution of the heat dome to the heatwave

**Fig. 2:**
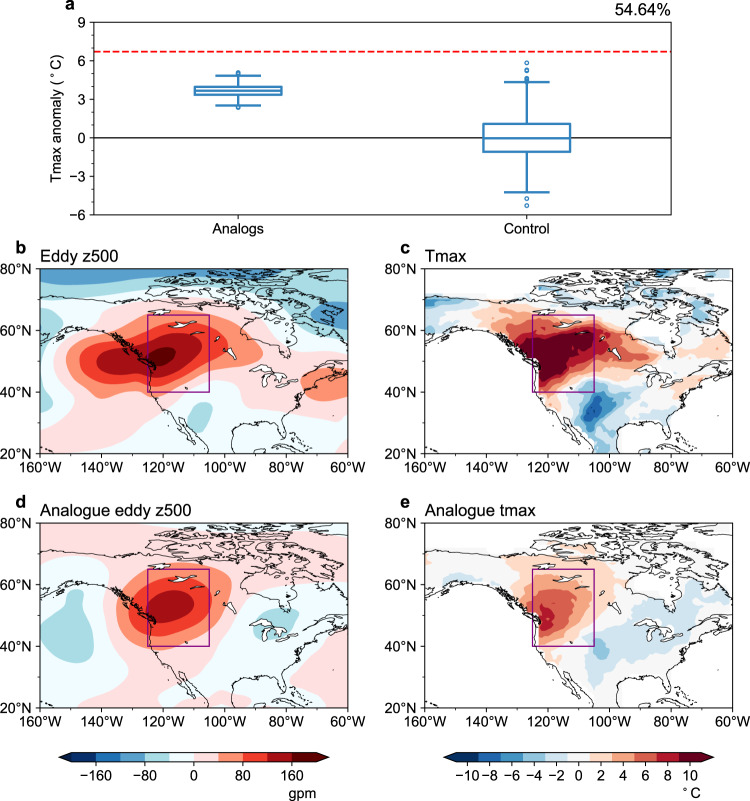
The contribution of the heat dome to 2021 heatwave over the Western North America. **a** The distribution of the maximum temperature anomalies (detrend, unit:  °C) for flow analog days generated using eddy geopotential height anomalies at 500 hPa from 27 June–3 July and randomly picked days (control) during 1959–2020. The three lines of boxes denote the 25th percentile, median and 75th percentile from bottom to top, respectively. The red line indicates the maximum temperature anomalies averaged from 27 June–3 July 2021. **b**, **c** Spatial distribution of the eddy geopotential height anomalies at 500 hPa (unit: gpm) and maximum temperature anomalies (unit:  °C) averaged from 27 June–3 July 2021. **d**, **e** is the same as **b**, **c** but for the randomly picked flow analog days during 1959–2020.

To quantify the contribution of anomalous high-pressure circulation to the heatwave, in reference to the hottest week (27 June–3 July 2021), we choose similar eddy geopotential height anomalies at 500 hPa (eddy z500 anomalies) during 1959–2020 over the WNA (purple box in Fig. 1c) using the flow analog method (see Methods). We compare the distribution of tmax anomalies for eddy z500 anomaly flow analog days and randomly picked days during 1959–2020 derived from ERA5 reanalysis in Fig. 2a. Compared to the randomly picked days (i.e., unconditioned on circulation), the tmax anomalies corresponding to similar heat dome circulations over the WNA are significantly higher, demonstrating the dominance of the heat dome on the heat extreme. The median of the tmax anomaly distribution on circulation analog days is 3.67 °C (with a 25–75 percentile range of 3.36 °C–3.97 °C), indicating that the heat dome can explain 54.64% of the observed temperature anomalies (6.71  °C).

We also carry out a sensitivity test on the domain size, circulation proxy, and duration of events (Supplementary Fig. 3). To test the spatial extent of the heat dome, the three domains, small domain (45°−60°N, 110°−125°W), middle domain (40°−65°N, 105°−125°W), and large domain (40°−65°N, 100°−130°W), are used to select analog days (Supplementary Fig. 3a). The contributions from the heat dome to extreme high-temperature anomalies are similar (approximately 50%) in all three cases, indicating that the estimation of circulation contribution is robust against the domain size. Specifically, the contribution from the heat dome would be slightly larger when using a smaller domain, in line with previous studies^22,30–32^. We use the middle domain in our analysis.

For the circulation proxy, tmax anomalies conditional on the eddy geopotential height anomalies at 300 hPa (eddy z300 anomalies) are larger than those conditional on other levels (including eddy z500, eddy z700, and eddy z850 anomalies), with a percentage contribution of 68.06% (Supplementary Fig. 3b). Since the lower levels are influenced more by the underlying surface, the percentage contributions of flow in the lower levels are generally smaller than those at higher levels.

We compare the heatwaves with different durations, including 3-day (29 June–1 July 2021), 5-day (28 June–2 July 2021) and 7-day heat events, which are the hottest periods in summer. The contributions of the heat dome are similar (approximately 50%), with the largest contribution seen for the 7-day heat event (Supplementary Fig. 3c). In addition, the sampling uncertainty is minimal for the longest event (consistent with ref. ^22^).

We further compare the eddy z500 and tmax anomalies of the hottest week observed in 2021 and the reconstructed results from flow analog days in Fig. 2b–e. It is evident that circulations over the WNA are similar (cf. Fig. 2b, d), but the temperature anomalies are significantly lower for analogs (cf. Fig. 2c, e). A previous study indicated that the atmospheric circulation of the 2021 heat event was not unique, while the corresponding temperatures were^12^. This result implies potentially inconsistent changes in circulation and temperature.

**Fig. 3:**
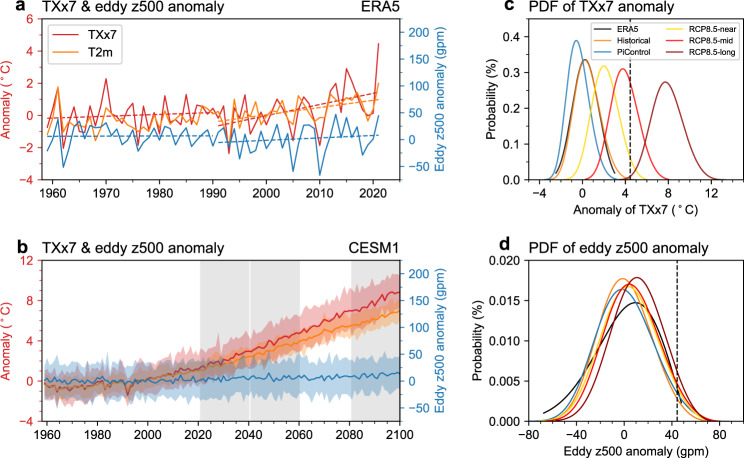
The time series and probability density function (PDF) distribution of *TXx7* anomalies and corresponding eddy geopotential height anomalies. Here, *TXx7* is defined as the annual summer (June-August) maxima of the 7-day running mean of the daily maximum temperature anomaly area-weighted averaged over Western North America (WNA). **a** The anomalies of annual summer *TXx7* (red solid line, unit:  °C) and corresponding eddy geopotential height anomalies (blue solid line, unit: gpm) at 500 hPa area-weighted averaged over the WNA during 1959–2021 for ERA5 reanalysis. The orange line denotes the summer mean 2 m mean temperature anomaly area-weighted averaged over the WNA. The dotted line denotes the trend of *TXx7* anomalies and corresponding eddy geopotential height anomalies. **b** is the same as **a** but for the CESM1 historical and RCP8.5 simulations during 1959–2100. The gray shadows denote the near-term (2021–2040), mid-term (2041–2060), and long-term (2081–2100). Solid lines represent the results of the ensemble mean; the light shades are for the 5th percentile to 95th percentile for the 40-member. **c** The PDF distribution of *TXx7* anomalies during 1991–2020 for ERA5 reanalysis (black curve), during 1991–2020 for 40-member realization in CESM1 historical simulation (orange curve), for 1620 years of preindustrial control simulation (blue curve), for RCP8.5 simulation in the near-term (yellow curve), mid-term (red curve) and long-term (dark red curve). The black vertical line indicates the *TXx7* anomalies of 2021 from ERA5. **d** is the same as **c** but for the PDF distribution of eddy geopotential height anomalies corresponding to *TXx7*. The black vertical line indicates the eddy geopotential height anomalies of the average of 27 June–3 July 2021 from ERA5.

We examine the long-term changes in extreme temperature and heat dome-like atmospheric circulation in Fig. 3a by showing the time series of the summer *TXx7* anomaly and the corresponding eddy z500 anomaly area-weighted averaged over the WNA. The temperature index exhibits an accelerated warming trend since 1990, while no similar trend is seen in the evolution of a heat dome-like circulation index. The intensification of hot extremes is accompanied by an increase in the summer mean temperature measured by t2 m. Although we do not see an evident intensification of heat dome-like circulation anomalies, we do find that the hot extremes intensified after the 1990s in association with accelerated background climate warming (Fig. 3a). This suggests that the hot extreme could increase with global warming faster than circulation.

We further examine the relationship of extreme temperature with the heat dome-like atmospheric circulation in the model world. The Community Earth System Model version 1 (CESM1) Large Ensemble^33^ from 1959 to 2100 with 40-member daily output is used in our study. The historical forcing of CMIP5 is applied until 2005, and the radiative forcing of RCP8.5 is applied from 2006 to 2100. We divide this period into the historical period (1959–2020) and future projection (2021-2100) under the RCP8.5 high emissions scenario. The model well simulates the spatial distribution of tmax anomalies of the summer hottest week and the associated circulation anomalies represented by eddy z500 anomalies (Supplementary Fig. 4), as well as the significant positive correlation between circulation and high temperature (Supplementary Fig. 5). The increasing trend of *TXx7* and its probability density function (PDF) distribution are also accurately reproduced (Supplementary Fig. 6a, b).

The changes in circulation and temperature in both the historical climate simulation and future climate projection of the CESM1 large ensemble are similar to the results of ERA5 (Fig. 3b), demonstrating the robustness of the results. The inconsistent changes between the heat dome-like circulation anomalies and hot extremes are also evident in the PDF distributions (Fig. 3c). In both preindustrial control simulations (viz. The concentration of CO_2_ was set to a preindustrial value and remained unchanged during the simulation) and historical climate simulation ensembles in the present period (1991–2020), the range of the distribution of the *TXx7* anomalies spans negative to positive values, and the latter includes the observed 2021 *TXx7* warm anomaly, indicating the role of internally generated atmospheric variability in the occurrence of heat events. There is an obvious rightward shift in the PDF of the *TXx7* anomalies in the historical simulation compared to the preindustrial control simulation, indicating that human influence has increased the probability of extreme warm events. Relative to the preindustrial control simulation, the best-estimate probability ratio (PR) and fraction of attributable risk (FAR) are 807 and ~1, respectively, for the present period of historical climate simulation (see Methods). In contrast to the extreme temperature, the circulations show indistinguishable distributions in the preindustrial control simulation and historical climate simulation (passing the Kolmogorov-Smirnoff (K-S) test with *p* value of 0.64) (Fig. 3d), indicating no significant change in the heat-dome-like atmospheric circulation.

In climate projections, the *TXx7* anomalies will continue to strengthen in the future under the RCP8.5 scenario, with an approximately 33% probability of a similar extreme event in the mid-term (2041–2060) projection, while in the long-term (2081–2100) projection, such extreme high-temperature events will become average (Fig. 3c). In contrast to the significantly increased probability of extreme warm events, the probability change of a heat dome-like circulation is weak. Although the PDF distributions of the circulation in the future projections are distinguished from the present period of historical climate simulation (*p* value ~0 for all K-S tests), the probability of extreme high-pressure circulation (i.e., larger than the observed 2021 anomaly) is 4.2%, 5.1%, and 6.1% in the near-term (2021–2040), mid-term and long-term projections, which is approximately 1.8, 2.2, and 2.6 times higher than the historical simulation (Fig. 3d), indicating the increased probability of the heat dome-like circulation change is also significant, albeit smaller than that of the temperature.

### Influence of background warming on the heatwave

**Fig. 4:**
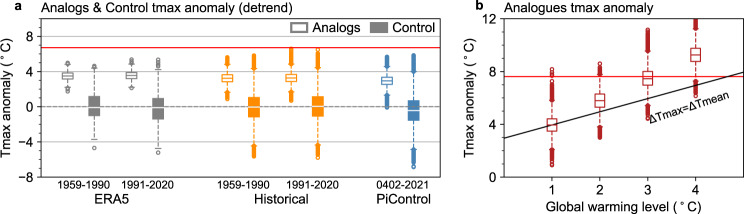
The intensities of hot extremes associated with similar heat domes increase faster than global mean temperature. **a** The distribution of the maximum temperature anomalies (detrend, unit:  °C) for flow analog days (analogs, unshaded) generated using eddy geopotential height anomalies at 500 hPa from 27 June–3 July and randomly picked days (control, shaded) for ERA5 reanalysis and CESM1 simulations. **b** The distribution of the maximum temperature anomalies (unit:  °C) for flow analog days in given levels of projected global warming. The black line corresponds to ∆Tmax = ∆Tmean, indicating the increase in extreme temperature is equal to global mean temperature. The red line indicates the maximum temperature anomalies of the average of 27 June–3 July 2021 from ERA5 in both figures.

The above analysis shows inconsistent long-term changes in circulation and temperature. Does this result indicate the impact of a heat dome on the heatwave would enhance under a warmer climate even though the heat dome itself remains unchanged? To address this question, we apply the flow analog method to two different historical periods: the earlier (1959–1990) and present (1991–2020) periods. In the ERA5 reanalysis, the tmax anomalies over the WNA are significantly higher in the present period (3.55 °C: 3.20–3.88 °C) than in the earlier period (3.49 °C: 3.16–3.84 °C) under similar heat dome circulations as those in 2021 (Fig. 4a, *p* < 0.05). Since the trend of temperature has been removed, it indicates that the intensities of hot extremes associated with similar heat domes have increased faster than background warming.

We further examine the increased impact of background warming on heatwaves under a specific circulation in climate models using the flow analog method (see Methods). The tmax anomalies under similar heat domes increase continuously from the preindustrial period (2.94 °C: 2.53–3.35 °C) to the earlier historical period (3.23 °C: 2.83–3.63 °C), and to the present period (3.26 °C: 2.87-3.66 °C) (Fig. 4a), indicating that anthropogenic forcing has increased the temperature under similar circulations, making heat events more likely to happen. Under given global warming levels, the tmax anomalies increase faster than the mean surface temperature under similar circulations (Fig. 4b). For example, when the global mean temperature increases from 1 °C to 3 °C, the tmax associated with similar heat domes increases by 3.5 °C, indicating the intensities of hot extremes associated with similar heat domes increase faster than background global warming.

**Fig. 5:**
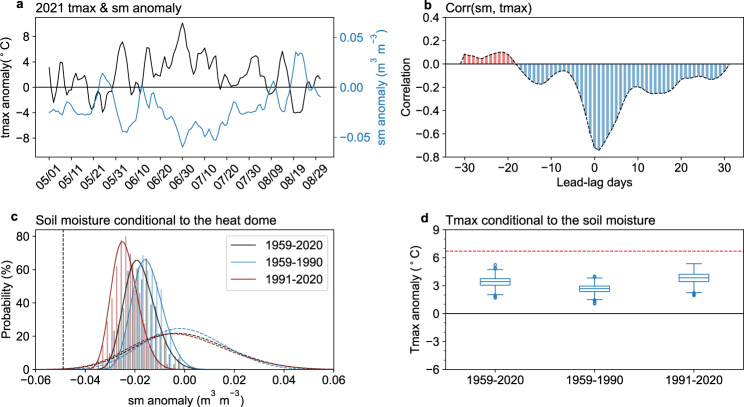
The influence of soil moisture on the temperature and circulation in ERA5 reanalysis. **a** The timeseries of maximum temperature (black, unit:  °C) and soil moisture (blue, unit: m^3^ m^−3^) anomalies (relative to the 1981–2010 average for each calendar day) during 1 May–31 August 2021. **b** The lead-lag correlation between maximum temperature and soil moisture anomalies in 2021. The negative values of the x-axis indicate that soil moisture leads to temperature. **c** Histogram (bars) and probability density functions (PDF, solid curve) of simultaneous soil moisture anomalies (unit: m^3^ m^−3^) under a similar circulation of 27 June–3 July 2021, and the dashed curves denote the soil moisture anomalies for randomly picked days. The dashed vertical line indicates the soil moisture from 27 June–3 July 2021. **d** The distribution of the maximum temperature anomalies (detrend, unit:  °C) for analogs days generated using detrended soil moisture anomalies from 27 June–3 July 2021 during 1959–2020, 1959–1990, and 1991–2020. The red line indicates the maximum temperature anomalies of the average of 27 June–3 July 2021 from ERA5.

Apart from the circulation, the hot extreme is also related to the soil moisture deficit, which can amplify heatwaves through land‒atmosphere feedbacks^34–37^. We examine the time evolutions of soil moisture and temperature and calculate the correlation coefficient between them (Fig. 5a, b) and find the largest simultaneous negative correlation between temperature and soil moisture, indicating that the temperature-soil moisture feedback plays an important role in the heatwave. We also compare the preceding month and simultaneous soil moisture conditional on the heat dome-like circulation from 27 June to 3 July 2021 with randomly picked-up days (see Methods), and find no evidence supporting that the preceding soil moisture is responsible for the formation of the heat dome (Supplementary Fig. 8a); instead, the heat dome is associated with simultaneous soil moisture deficits, reflecting a strong feedback between them (Fig. 5c). The dry soil moisture can enhance positive high-pressure anomalies at 500 hPa and make it more persistent^38^; meanwhile, the descending motion under high pressure can result in less rainfall and less cloud cover, which facilitates the decrease in soil moisture.

We also apply the flow-analog method to soil moisture by selecting soil moisture anomalies similar to each day from 27 June to 3 July 2021 (see Methods). We find that the soil moisture-temperature feedback over the WNA contributed 50.6% to the high temperature (Fig. 5d), indicating the important effect of soil moisture-temperature feedback on the hot extreme. The contributions of soil moisture and the heat dome are comparable, and the sum of the two contributions is not equal to 100% since there is an interconnection between them, i.e., drier soil moisture is associated with anomalous anticyclonic circulation, and vice versa. We compare the probability density distributions of soil moisture associated with similar heat domes in three time periods, including 1959–2020, 1959–1990, and 1991–2020 (Fig. 5c). We see a drier tendency in soil moisture during recent decades (Supplementary Fig. 8b), which is associated with an enhanced soil moisture-temperature feedback (Fig. 5d). Hence, the soil moisture-atmosphere feedback partly explains why the intensities of hot extremes associated with similar heat domes increase faster than background global warming.

The above analyses demonstrate that even if the intensity of the heat dome-like circulation anomalies remains unchanged, background warming and the enhanced soil moisture-atmosphere feedback would still lead to stronger extreme temperatures. Moreover, in future climate projections, the probability of extreme high-pressure circulation will increase, albeit with a weaker amplitude than the temperature (Fig. 3d), which will also be favorable for a further enhancement of extremely high temperatures.

**Fig. 6:**
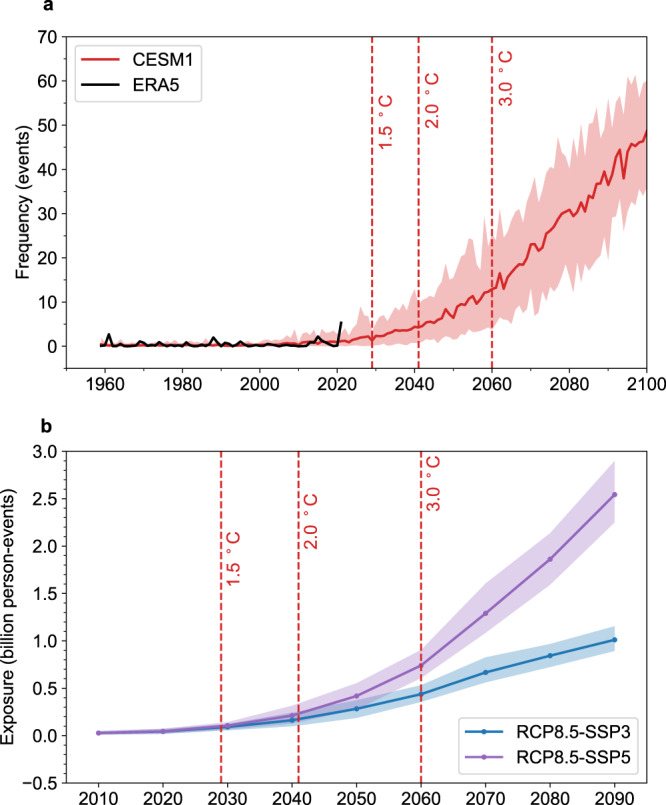
The frequency of 2021-like heatwaves and population exposure to heatwaves. **a** The heatwave frequency (unit: events) of ERA5 reanalysis (black) and CESM1 historical and RCP8.5 simulations (red) area-weighted averaged over Western North America (WNA). **b** Population exposure to heatwaves across the WNA through the 21st century in the integrated scenario: RCP8.5-SSP3 with rapid growth in greenhouse gas emissions and low growth in population (blue, unit: billion person-events), RCP8.5-SSP5 with rapid growth in greenhouse gas emissions and higher growth in population (purple). The 1.5 °C, 2 °C, and 3 °C global warming levels are reached in 2029, 2041, and 2060, respectively.

How will the 2021-like heat extreme change in the future? In the observations, the *TXx7* of the 2021 heatwave reached 7.61 °C. Using this temperature as the threshold, we make projections of the frequency change of the 2021-like extreme heat over the WNA (see Methods). Under the RCP8.5 scenario, by the end of the 21st century, the frequency of similar heat events will increase by 40-fold compared to that in 2021 (Fig. 6a). To mirror the policy discourse surrounding the Paris Agreement targets of 1.5 °C and “well below 2 °C”, we also assess changes at global warming levels of 1.5 °C, 2 °C and 3 °C (see Methods). In the 1.5 °C warmer world, the frequency of similar heat events could be doubled compared to 2021, while in the 2.0 °C and 3.0 °C warmer world, the frequency could increase by 3-fold and 10-fold, respectively, with more recurring heat events. Hence, if we limit global warming to 1.5 °C instead of 2 °C (3.0 °C) (see Methods), ~65% (92%) of the increase in the frequency of the 2021-like heat extreme could be avoided in the WNA.

For the exposure of the population to extreme heat events (see Methods), under the RCP8.5-SSP3 scenario with rapid growth in greenhouse gas emissions and low population growth^39^, the population exposure will nearly triple from 0.03 billion person-events in the 2010s to 0.09 billion person-events in a 1.5 °C warmer world (Fig. 6b). In 2 °C and 3 °C warmer worlds, the population exposure is projected to increase to 0.16 billion and 0.44 billion person-events, respectively, nearly 6 and 16 times the baseline period. Under the RCP8.5-SSP5 scenario with rapid growth in greenhouse gas emissions and higher population growth, the population exposure is projected to increase by 3-fold, 7-fold, and 26-fold relative to the baseline period in the 1.5 °C, 2 °C and 3 °C warmer worlds (Fig. 6b). Since the population in North America increases faster under the SSP5 scenario than under the SSP3 scenario^39^, the population exposure is higher under the RCP8.5-SSP5 scenario. Hence, if we limit global warming to 1.5 °C instead of 2 °C (3.0 °C), the 0.5 °C (1.5 °C) less warming would reduce the population exposures to 2021-like heat extreme in the WNA by ~53% (89%) under the RCP8.5-SSP5 scenario.

## Discussion

The Western North America experienced nearly a week of extremely high temperatures, from 27 June to 3 July 2021, accompanied by an anomalous high pressure — a heat dome. Using the flow analog method, we show that the heat dome over the WNA contributed over 50% of the magnitude of the high temperature. Applying the flow analog method to soil moisture also finds strong feedback to heat extremes. In the observations, the intensities of high-temperature extremes associated with similar heat dome-like atmospheric circulations increase faster than background global warming partly due to the feedback of soil moisture. Such a relationship between hot extremes and global mean temperature under heat dome-like atmospheric circulations is also evident in historical climate simulations and further warming projections. The probability of 2021-like heat extremes is projected to increase due to the background warming, the enhanced soil moisture-atmosphere feedback and the weak but still significantly increased probability of the heat dome-like circulation. Populations exposed to heatwaves in the WNA will increase, especially under scenarios such as RCP8-SSP5. The 0.5 °C (1.5 °C) lower warming will help avoid 53% (89%) of the increase in population exposure to the 2021-like heat extreme in the WNA under the RCP8-SSP5 scenario.

While the flow analog method has been demonstrated to be a useful method in quantifying the role of atmospheric circulation in extreme events, we also acknowledge some of its limitations. First, the percentage contribution of flow analog could be affected by domain size, circulation proxy, and the duration of the heatwave. While our sensitivity tests indicate qualitatively consistent results, quantitative differences still exist. Second, observations are always limited, and analogs may not fully reproduce the circulation of the extreme event, which is referred to as “sampling uncertainty” in the climate attribution community^22^. Third, the role of soil moisture feedback and other feedback processes, such as snow/ice albedo feedback, are not considered in the selection of similar circulations^22,40^. Fourth, the 20 analogs in each day may be related to another day because of the persistence of the atmospheric circulation, which may affect the effective sizes of the samples^22^. Hence, a comparison of flow analog analysis to results based on different research methods is encouraged.

To examine whether there is a maximum effect of the heat dome in this region, we analyze the influence of the heat dome using unprecedented model events. For unprecedented heat events with *TXx7* exceeding 6, 8, and 12 standard deviations ($\sigma$) under the RCP8.5 scenario (see Methods), the heat dome can explain 53.86%, 50.24%, and 44.77%, respectively (Supplementary Fig. 3d). Although the percentage contribution of atmospheric circulation to the heatwave slightly decreases with the magnitude of events, we cannot find a maximum effect of the heat dome in this region.

We acknowledge the potential model dependence of the projection. CESM1 has an equilibrium climate sensitivity of 4.10  °C^41^, which is slightly higher than the likely range of 2.5  °C−4  °C assessed by IPCC AR6^3^, leading to potentially stronger warming of future projection. Although using global warming levels in our risk analysis has bypassed the impact of climate sensitivity as recommended^42^, the potential impact of climate sensitivity on the long-term extreme high-temperature projection warrants further investigation.

We have employed the flow analog method for the circulation and soil moisture. We highlight the important role of circulation, while the feedback of soil moisture is a complementary mechanism as the previous study^29^. We acknowledge that the atmosphere, clouds, and soil moisture are tightly coupled in the real world. The change in cloud cover is associated with high pressure and soil moisture, as the anticyclic circulation could cause low relative humidity, high temperature and reduced cloudiness^43^, which may initially stimulate soil evaporation^43,44^. When the soil moisture dries below a critical threshold, evaporation starts to decrease, and the reduction in evapotranspiration further reduces cloud cover and rainfall^45–47^. Soil moisture can provide feedback on circulation through cloud cover and radiation changes^37,48,49^. The interaction of cloud cover, high pressure and soil moisture needs to be investigated.

Finally, previous studies found that the probability ratio of extreme events, including heat extremes, increases nonlinearly with global mean temperature^50^, and our study highlights that the intensity of hot extremes increases faster than background global warming under similar circulations. According to IPCC AR6, the observed increase in extreme heat events is significant in Western North America^3^. Nevertheless, although some studies have indicated that more anticyclones have occurred in some mid-latitudes in recent decades^26^, there is no significant trend in anticyclonic circulation in the Northern Hemisphere during boreal summer^51,52^. This also supports our results in less change in circulation than temperature, but its dynamical causes need to be further investigated.

## Methods

### Observations

Observations and reanalysis products are used in this study. The observational daily maximum temperature (tmax) data during 2021 are from the Global Historical Climatology Network Daily database (GHCN-D)^53^. The daily mean data, including geopotential height at 500 hPa (z500), 300 hPa (z300), 700 hPa (z700), 850 hPa (z850) and soil moisture from 1959 to 2021, are obtained from averaging 4 time hourly data in each day from the European Centre for Medium-Range Weather Forecasts (ERA5)^54^, while daily tmax is obtained from the highest values of the 24-hour data. Daily anomalies are calculated relative to the 1981–2010 average for each calendar day. In addition, the monthly 2 m mean temperature (t2 m) from ERA5 is used in the study.

### Numerical simulations

The daily z500 and tmax simulated by the historical, RCP8.5, and preindustrial control simulations (piControl) from the Community Earth System Model version 1 (CESM1) Large Ensemble Project^33^ are analyzed. Monthly t2 m of historical and RCP8.5 simulation are also used in this study. It includes a 40–member ensemble for 1920–2100. The historical forcing of CMIP5 is applied until 2005, and the radiative forcing of RCP8.5 is applied from 2006 to 2100. Since the historical simulation only covers the period up to 2005, we extend the historical simulation to 2021 by splicing the 2006–2021 period from the RCP8.5 simulation. We divide RCP8.5 (2021–2100) into the near-term (2021–2040), mid-term (2041–2060) and long-term (2081–2100). The preindustrial control simulation experiment adopts the data from years 402 to 2021. In addition, the daily tmax from model years 850 to 2005 simulated by the CESM1 last millennium ensemble (LME), including 12 members^55^, is also used to examine the extreme event from a past millennial perspective. All data are gridded to a common grid of 1° × 1°.

To investigate the effect of heatwaves on population exposure, we also use population projection distributions under different shared socioeconomic pathways (SSPs) (SSP3 and SSP5) between 2010 and 2100^56,57^. Since population projection data are provided at a spatial resolution of 1/8° × 1/8°, we add up the total population in the 1/8° population grid within a large grid of 1° × 1° to keep it the same grid as the climate dataset^58^.

### Definition of extreme index

The summer 2021 heatwave mainly affected Western North America (WNA, 40°−65°N, 105°−125°W, purple box in Fig. 1c, Supplementary Fig. 1 and Supplementary Fig. 2). As the heat event lasted for approximately a week (Fig. 1a), the index *TXx7* is analyzed, which is defined as the annual summer (June–August) maxima of the 7-day running mean of the daily maximum temperature anomaly area-weighted averaged over the WNA, i.e., the hottest week of the summer per year^59^.

The ensemble mean of the CESM1 historical simulation well simulates the spatial distribution of the tmax anomalies of the summer hottest week and the associated circulation anomalies represented by eddy z500 anomalies compared to ERA5 (Supplementary Fig. 4). The positive correlation is significant in CESM1 simulations, including historical and future periods (Supplementary Fig. 5), which is the basis of quantifying the contribution of circulation to the heatwave. In terms of statistical characteristics, CESM1 reproduces the long-term evolution of *TXx7* anomalies (Supplementary Fig. 6a), and the probability density function (PDF) distribution (Supplementary Fig. 6b) passes the Kolmogorov-Smirnoff (K-S) test with a *p* value of 0.26, indicating that CESM1 can capture the characteristics of heatwaves. The PDF is fitted with a generalized extreme value (GEV) distribution.

### Bias correction method

The corrected *TXx7* simulated by the CESM1-LME and the CESM1 historical simulation are estimated by a bias correction method^60,61^ using the observed *TXx7*, following Eq. (1).

1 $${{TXx}7}_{i}^{{{{{{\rm{OBS}}}}}}}=C+{{TXx}7}_{i}^{{{{{{\rm{Model}}}}}}}.$$

where ${{TXx}7}_{i}^{{{{{{\rm{OBS}}}}}}}$ and ${{TXx}7}_{i}^{{{{{{\rm{Model}}}}}}}$ represent the *TXx7* for year *i* in the observation and simulation, respectively, and the constant offset *C* is calculated following Eq. (2).

2 $$C=\frac{1}{n}\mathop{\sum }\limits_{j=1}^{n}{{TXx}7}_{j}^{{{{{{\rm{OBS}}}}}}}-\frac{1}{n}\mathop{\sum }\limits_{j=1}^{n}{{TXx}7}_{j}^{{{{{{\rm{Model}}}}}}}.$$

where *C* represents the average difference between the observation and simulation during the 25-year (*n*) reference period (1981–2005). After correction, the PDF distributions of *TXx7* for CESM1-LME are comparable to the ERA5 reanalysis (Supplementary Fig. 7).

### Flow analog method

To quantify the atmospheric circulation contribution to the extreme high temperature over the WNA, we apply the *flow analog method*^21–23^, with the circulation proxy eddy geopotential height anomalies at 500 hPa (eddy z500 anomalies), where eddy z500 is calculated by removing the zonal mean from the geopotential height at 500 hPa^24^. For each day during this event (27 June-3 July) with an anomalous anticyclone located over the WNA, 20 best analog days with the most similar eddy z500 anomaly field over the WNA are selected within a 61-day ($\pm$30 days) moving window centered on it from other years. The similarity is determined by the Euclidean distance between the observation and the analog circulation maps over the WNA. The similarity can also be measured by the pattern correlation coefficient between the analogs and the event. The two measures of similarity yield qualitatively similar results^25^. After, the probability distribution of tmax anomalies conditional on the circulation is reconstructed in three steps: (i) For each day, one of the 20 best analog days is randomly selected and then a sequence of tmax anomalies during the heat event is combined; (ii) the average of the sequence of tmax anomalies is calculated; (iii) by repeating the above two steps 1000 times, the probability distribution of tmax anomalies conditional on the 2021 heatwave circulation is obtained. For comparison, the distribution of tmax anomalies is reconstructed from a randomly selected series of days of the same length as the 2021 event to serve as a control group, which represents tmax anomalies unconditioned on circulations. The temperature is detrended before analysis for removing the influence of global warming.

Similar to the ERA5 reanalysis, we also apply the flow analog method to the data of CESM1 historical and preindustrial control simulations to assess human influence. Notably, as the length of the dataset affects the similarity of the selected circulation^22^, the preindustrial control simulation is divided into 54 groups (each with a length of 30 years) by applying the flow analog method to each group separately. In addition, the timing of 1 °C, 2 °C, 3 °C and 4 °C warming relative to the pre-industrial level (1850–1920) is estimated according to the evolution of global mean near-surface air temperature with an 11-year running window. In given global warming levels, the probability distribution of tmax anomalies from circulation analogs is derived using flow analog method.

To test the unprecedented extreme events in the CESM1 model, we pick up the model events with *TXx7* exceeding 6, 8 and 12 standard deviations ($\sigma$) under the RCP8.5 scenario compared with *TXx7* in heatwave 2021 exceeding ~5$\sigma$. First, the year in which *Txx7* exceeds 6, 8 and 12$\sigma$ for the first time in each member and the corresponding dates are picked out. Second, for 40-member extreme heat events, the flow analog method is applied based on eddy z500 anomalies for ERA5 reanalysis during 1959-2020. Finally, the contribution of circulation could be derived from the median of the probability distribution of maximum temperature anomalies for multiple members.

To investigate the effect of soil moisture on the heat dome, we select the preceding month and simultaneous soil moisture anomalies conditional on the heat dome-like circulation from 27 June to 3 July 2021. Specifically, for each day during this event (27 June-3 July 2021), we select one of 20 best analogs with the most similar eddy z500 anomaly field over the WNA, obtain the soil moisture anomalies of this date, and then average the soil moisture of 7 days. By repeating the above steps 1000 times, the probability distribution of simultaneous soil moisture anomalies conditional on the heat dome-like circulation from 27 June to 3 July 2021 is obtained. The probability distribution of preceding soil moisture anomalies is obtained similarly, but the mean of soil moisture for the first 30 days of the selected date among the 20 best analogs should be calculated. For comparison, soil moisture anomalies of randomly picked days are set to the control group.

To quantify the contribution of soil moisture anomalies to the heatwave, we select the 20 best analog days with the most similar soil moisture anomaly fields over the WNA, that is, replace the circulation proxy with soil moisture anomalies, and then obtain the probability distribution of tmax anomalies conditional on the soil moisture of the 2021 case through randomly picked analogs.

### The estimate of probability ratio (PR) and fraction of attributable risk (FAR)

To compare the probability of hot extremes occurring, the probability ratio (PR) and fraction of attributable risk (FAR) are calculated following Eq. (3) and Eq. (4)^6,50,62^.

3 $${{{{{\rm{PR}}}}}}=\frac{{P}_{{{{{\rm{historical}}}}}}}{{P}_{{{{{\rm{piControl}}}}}}}.$$

4 $${{{{{\rm{FAR}}}}}}=1-\frac{{P}_{{{{{\rm{piControl}}}}}}}{{P}_{{{{{\rm{historical}}}}}}}.$$

where ${P}_{{{{{\rm{historical}}}}}}$ is the probability of the hot extreme occurring in the historical simulation and ${P}_{{{{{\rm{piControl}}}}}}$ is the probability of the hot extreme occurring in the preindustrial control simulation. The best estimate values are estimated by the median using the bootstrap method.

### Frequency and population exposure to extremes at different warming levels

Using the *TXx7* of 2021 as the threshold, the number of times that this threshold is exceeded on a 7-day running mean in summer each year at each grid is calculated as the frequency.

Considering both climate and population projections, population exposure is calculated as the summer number of events (i.e., frequency) multiplied by the number of people exposed at each grid^58,63,64^. The population is projected per decade; for this reason, we calculate the decadal average for the frequency of heatwaves.

To compare the effect of warming levels on the frequency of heatwaves and population exposure, the 1.5 °C, 2 °C, and 3 °C warming periods are derived. According to the evolution of global mean near-surface air temperature with an 11-year running window of CESM1 historical and RCP8.5 simulations^65^, the timing of 1.5 °C, 2 °C and 3 °C warming relative to the preindustrial level (1850–1920) is 2029 (2024–2034), 2041 (2036–2046) and 2060 (2055–2065), respectively. The frequency of the 1.5 °C warming world is an average of 11 years during the warming period and the same for the 2 °C and 3 °C warming worlds, while the population exposure of the 1.5 °C, 2 °C and 3 °C warming worlds are estimated using the 2030 s, 2040 s, and 2060 s population exposures to heatwave, respectively.

### Avoided impacts

The impacts of extreme heat events that are avoided at 1.5 °C compared with 2 °C warmer climates are investigated using Eq. (5)^66^.

5 $${{{{{\rm{AI}}}}}}=\frac{{{{{{\rm{C}}}}}}2.0-{{{{{\rm{C}}}}}}1.5}{{{{{{\rm{C}}}}}}2.0}\times 100\%.$$

where AI is avoided impacts and C1.5 and C2.0 are the changes in the 1.5 °C and 2 °C warming climate compared with the present day. The avoided impacts of 1.5 °C warmer climates compared with 3 °C warmer climates are also calculated by Eq. (5), but C2.0 is replaced by C3.0, representing the changes in the 3 °C warmer climates compared with the present day.

## Supplementary information

Supplementary Information

## Data Availability

The data that support the findings of this study are freely available. CESM1 model data are from the National Center for Atmospheric Research [https://www.earthsystemgrid.org/dataset/ucar.cgd.ccsm4.output.html/]. Observational GHCN-D data are from the National Oceanic and Atmospheric Administration [https://www.ncei.noaa.gov/products/land-based-station/global-historical-climatology-network-daily/]. ERA5 reanalysis data are from the European Center for Medium Range Weather Forecasts [https://cds.climate.copernicus.eu/cdsapp#!/search?type=dataset]. The key processes data used in this study are available in the zenodo database [10.5281/zenodo.7652965].

## Code availability

The data in this study are analyzed mainly in Python (https://www.python.org/) with a few in the NCAR Command Language (NCL; http://www.ncl.ucar.edu/). Program to compute analogs available online at https://a2c2.lsce.ipsl.fr/index.php/licences/file/castf90?id=3 (Thanks to Aglaé Jézéquel, Pascal Yiou and Sabine Radanovics). Key codes used in this study are available in Zenodo via 10.5281/zenodo.7652965.
