# Supplementary material for: Increased impact of heat domes on 2021-like heat extremes in North America under global warming
Source: Nat Commun. 2023 Mar 27;14:1690. doi: 10.1038/s41467-023-37309-y (PMC10042826; doi:10.1038/s41467-023-37309-y)
Supplement: Supplementary file 1 — Supplementary Information [file 41467_2023_37309_MOESM1_ESM.pdf]

Increased impact of heat domes on 2021-like heat extremes in North America under global warming

Xing Zhang[1,2], Tianjun Zhou[1,2*], Wenxia Zhang[1], Liwen Ren[3], Jie Jiang[1], Shuai Hu[1], Meng Zuo[1], Lixia Zhang[1], Wenmin Man[1]

1. State Key Laboratory of Numerical Modeling for Atmospheric Sciences and Geophysical Fluid Dynamics (LASG), Institute of Atmospheric Physics, Chinese Academy of Sciences, Beijing 100029, China
2. University of Chinese Academy of Sciences, Beijing 100049, China
3. China Meteorological Administration, Beijing 100081, China

The file contains:

- Supplementary Figures 1-9

[*] Corresponding author: Tianjun Zhou (zhoutj@lasg.iap.ac.cn)

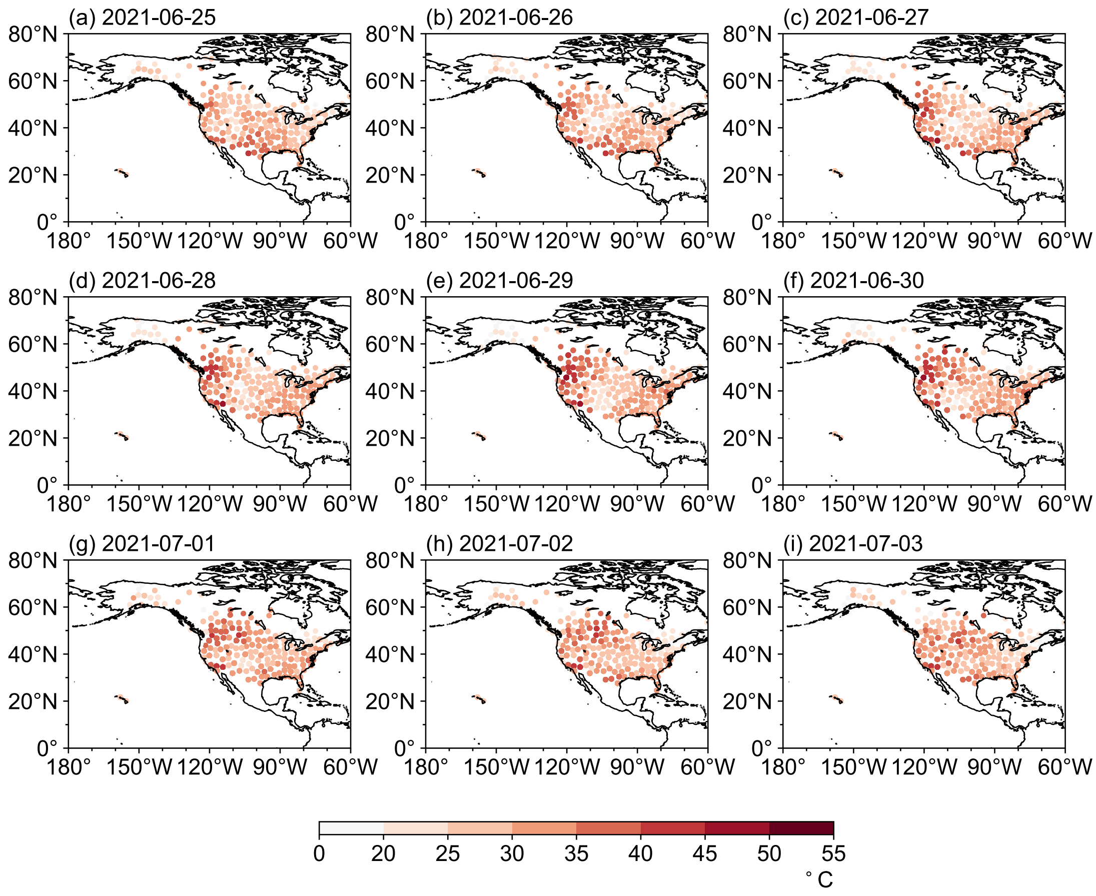

Supplementary Fig. 1 Daily maximum temperature (unit: °C) during 25 June-3 July over North America for the Global Historical Climatology Network daily (GHCN-D). (a)-(i) From 25 June to 3 July.

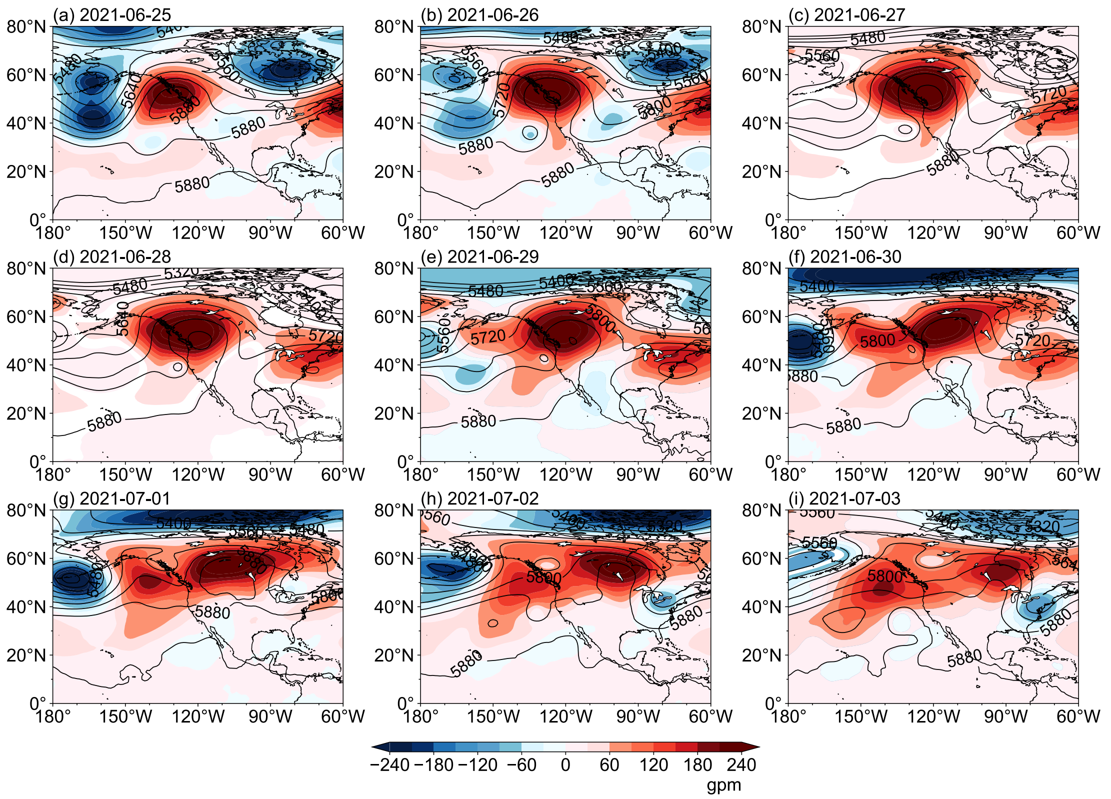

Supplementary Fig. 2 Daily geopotential height (contour, unit: gpm) at 500 hPa and its anomalies (shading) during 25 June-3 July over North America for ERA5 reanalysis. (a)-(i) From 25 June to 3 July.

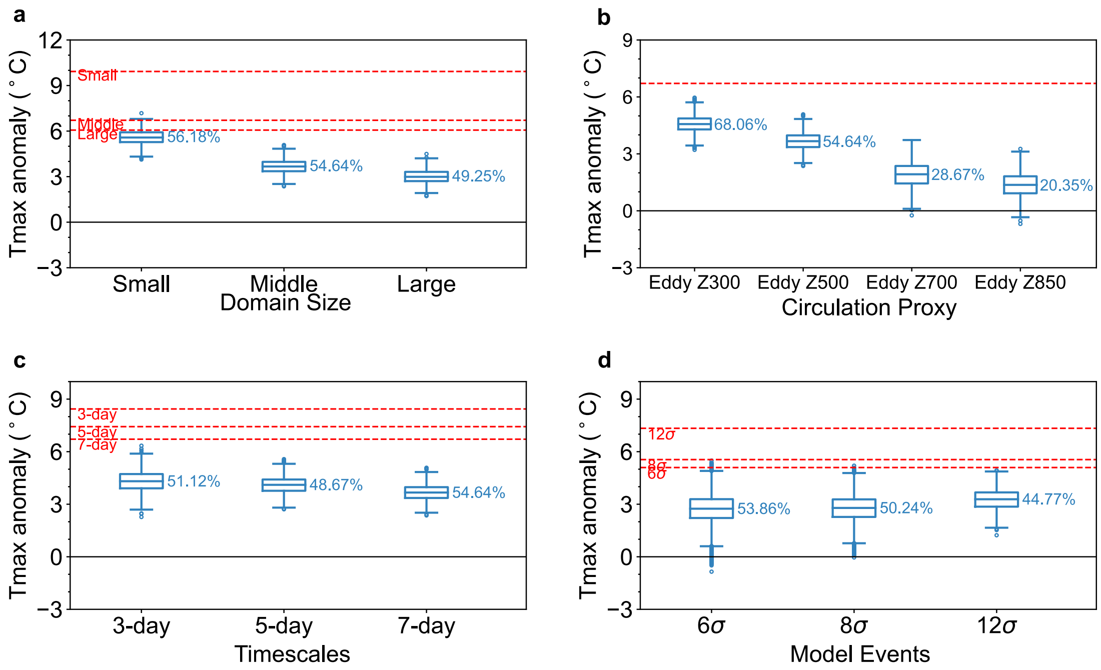

Supplementary Fig. 3 The distribution of maximum temperature anomalies conditional on the heatwave circulation during 1959-2020. (a) Using different sizes of the domain: small domain (45°-60°N, 110°-125°W), middle domain (40°-65°N, 105°-125°W), large domain (40°-65°N, 100°-130°W). (b) Using different circulation proxies: eddy geopotential height anomalies at 300 hPa, 500 hPa, 700 hPa and 850 hPa. (c) Different timescales of the heatwave: 3 days, 5 days and 7 days. In (a)-(c), the temperature anomalies are conditional on the 2021 heatwave circulation, and the observed event intensity is shown as a red line. (d) The circulation contribution to the unprecedented model events with *TXx7* exceeding 6, 8 and 12 standard deviations (σ) in the CESM1 RCP8.5 simulation (see Methods). *TXx7* is defined as the annual summer (June-August) maxima of the 7-day running mean of the daily maximum temperature anomaly area-weighted averaged over the Western North America. The numbers on the right-hand side of the box-and-whisker diagram represent the percentage contribution of the circulation to temperature.

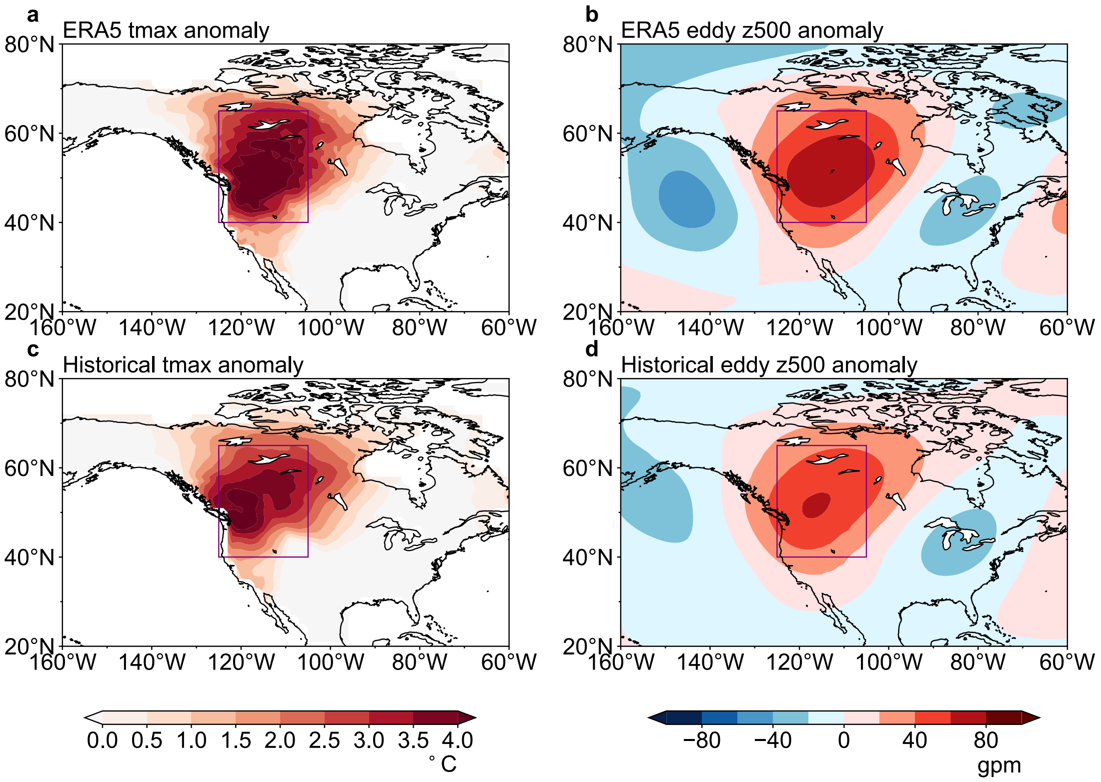

Supplementary Fig. 4 Spatial distribution of maximum temperature anomalies and eddy geopotential height anomalies at 500 hPa corresponding to *TXx7* of annual summer for ERA5 reanalysis and CESM1 historical simulation. (a)-(b) For ERA5 reanalysis. (c)-(d) For CESM1 historical simulation.

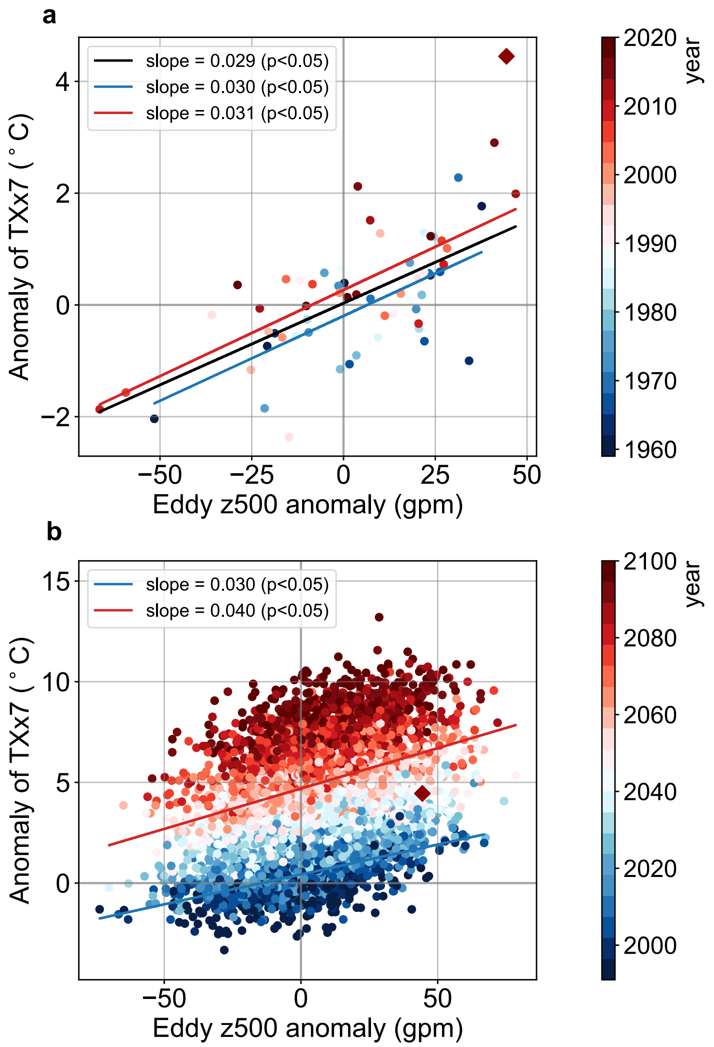

Supplementary Fig. 5 The scatter of *TXx7* and corresponding eddy geopotential height anomalies at 500 hPa anomalies over the Western North America (WNA). (a) ERA5 reanalysis during 1959-2021, colored by year. The red diamond represents the 2021 heat event. The black, blue and red lines represent the regression over 1959-2020, 1959-1990 and 1991-2020, respectively. (b) is the same as for (a), but for the CESM1 simulation. The blue and red lines represent the regression over 1959-2020 and 2021-2100.

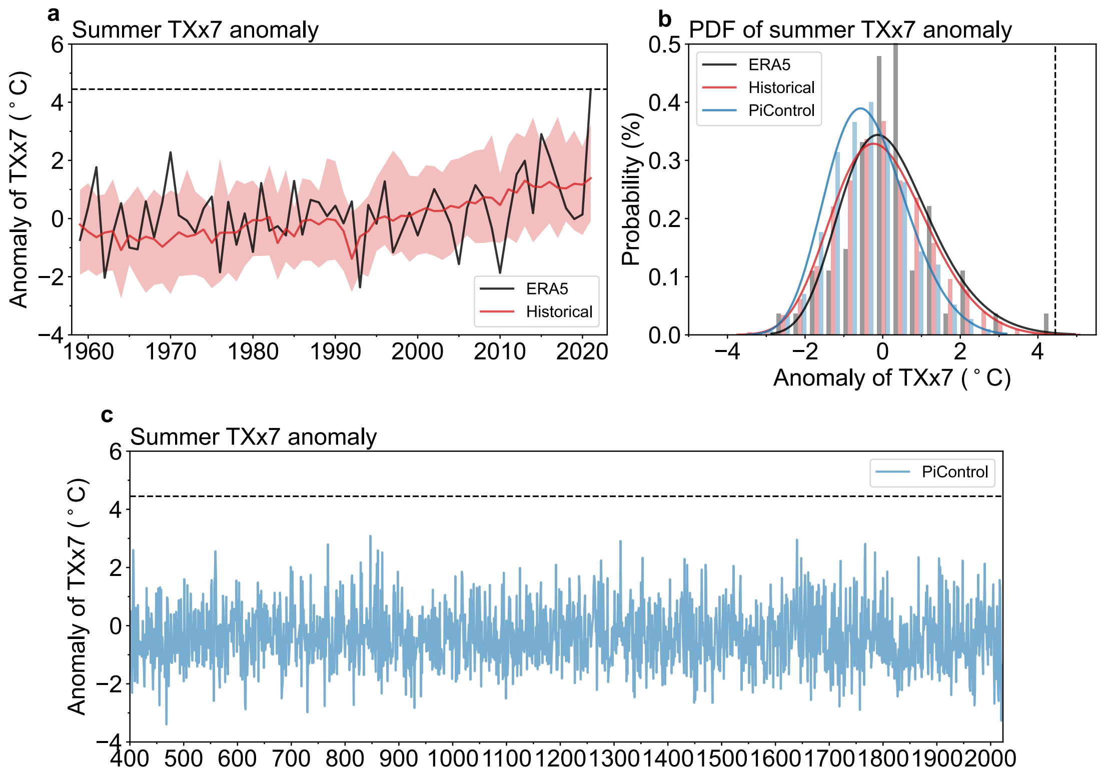

Supplementary Fig. 6 The time series and probability density function (PDF) distribution of *TXx7* anomalies. (a) The time series of *TXx7* anomalies of annual summer during 1959-2021 for ERA5 reanalysis and 40-member realization of CESM1 historical simulation. Solid lines represent the results of the ensemble mean; the light shades are for the 5th percentile to 95th percentile for the 40-member. (b) Histogram (bars) and PDF (curve) of summer *TXx7* anomalies averaged over the WNA during 1959-2021 for ERA5 (black bars and curve), 40-member realization in CESM1 historical simulation (red bars and curve) and 1620-year of preindustrial control (piControl) simulation (blue bars and curve). The black vertical line indicates the *TXx7* anomalies of 2021 from ERA5. (c) is the same as (a) but for the preindustrial control simulation.

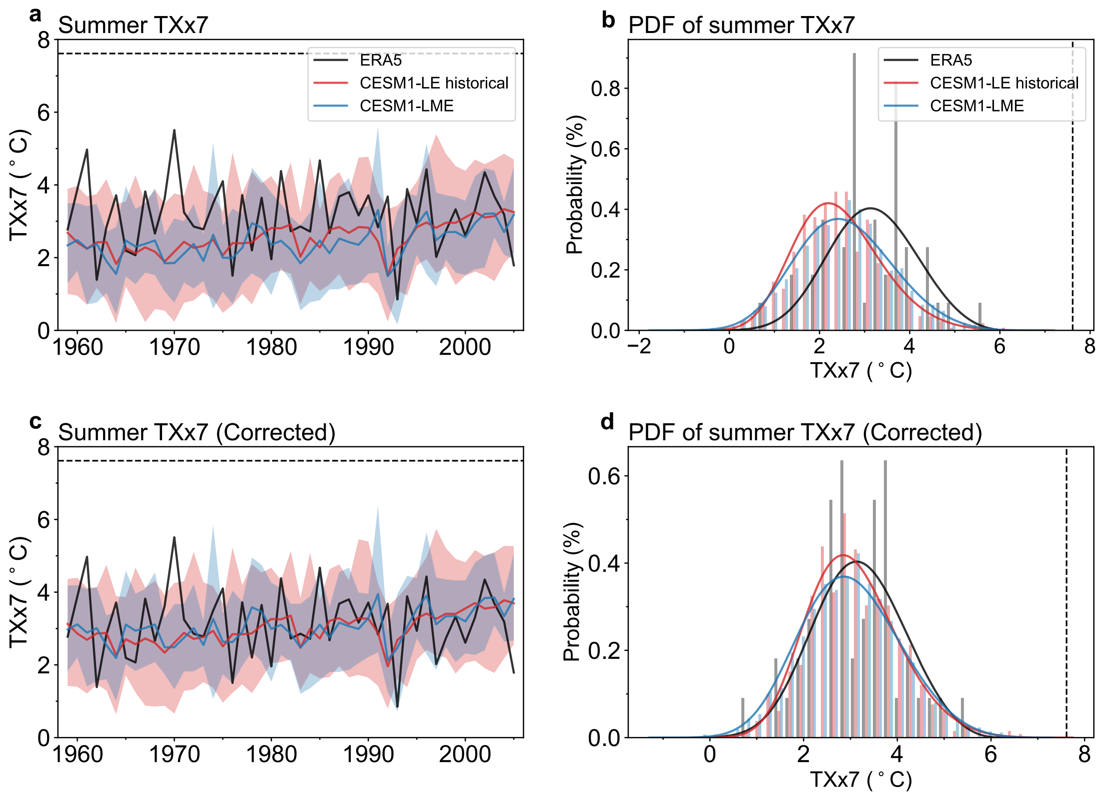

Supplementary Fig. 7 The time series and probability density function (PDF) distribution of *TXx7*. (a) Time series of *TXx7* of annual summer for ERA5 reanalysis, CESM1 historical simulation and CESM1 last millennium ensemble (LME) simulation from 1959-2005. Solid lines represent the results of the ensemble mean; the light shades represent the 5th percentile to the 95th percentile for multiple members. (b) Histogram (bars) and PDF (curve) of summer *TXx7* averaged over the WNA during 1959-2005 for ERA5 (black bars and curve), 40-member realization in CESM1 historical simulation (red bars and curve) and 12-member realization in CESM1 LME simulation (blue bars and curve). (c) and (d) are the same as (a) and (b), but the index *TXx7* is corrected using a bias correction method (see Methods).

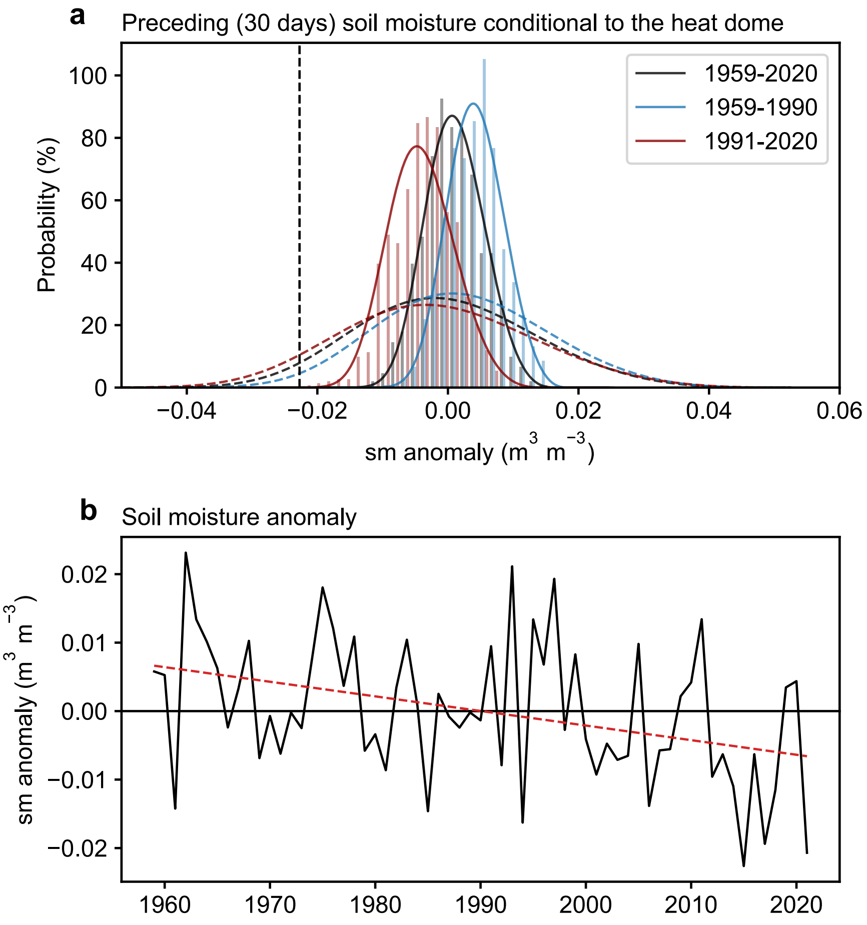

Supplementary Fig. 8 The effect of soil moisture on the heatwave. (a) Histogram (bars) and probability density function (PDF) (solid curve) of preceding (30 days - leading) soil moisture anomalies (unit: m^3 m^{-3}) under a similar circulation of 27 June-3 July 2021, the dashed curves denote the PDF of soil moisture anomalies for randomly picked days. (b) The timeseries of soil moisture anomalies and its trend averaged from May to August in 1959-2021.
